# Proteomic Analysis of *Lactococcus lactis* FM03 Under Prolonged Alkaline Stress

**DOI:** 10.1111/1462-2920.70381

**Published:** 2026-07-17

**Authors:** Tamara A. L. Bendig, Tjakko Abee, Sjef Boeren, Eddy J. Smid, Oscar van Mastrigt

**Affiliations:** ^1^ Food Microbiology Wageningen University and Research Wageningen the Netherlands; ^2^ Laboratory of Biochemistry Wageningen University and Research Wageningen the Netherlands

**Keywords:** high pH, lactic acid bacteria, physiology, proteomics, stress response

## Abstract

Lactococci are best known to thrive in acidic conditions. While their physiology in an acidic environment is well researched, their stress response towards an alkaline environment remains to be explored. In this study, we habituated *Lactococcus lactis* FM03 to pH 6, 7 and 8 in chemostats with a fixed dilution rate of 0.2 h^−1^ and defined the alkaline‐induced stress proteome. Based on an enrichment analysis of GO‐terms, we identified key physiological adaptations, including cell wall reinforcement, an increase in the uptake and metabolism of peptides and amino acids, as well as elevated levels of translation‐related proteins. Transmission electron microscopy revealed a doubling in cell wall thickness at pH 8 compared with pH 6, and we measured higher release of multiple amino acids in the supernatant at pH 8. Further, the compatible solute uptake system for glycine betaine (OpuA) was upregulated, indicating osmotic stress conceivably linked to low cyclic‐di‐AMP levels. Selected global stress proteins were upregulated at pH 8 (GroEL, HtrA, FtsH), and RelA increased, suggesting the activation of the stringent response, whereas acid resistance systems (F_0_F_1_‐ATPases, ADI, GAD) decreased. Together, this work defined a distinct alkaline stress remodelling of the proteome in *L. lactis* FM03 under sustained alkaline growth.

## Graphical abstract

Overview of alkaline resistance mechanisms in *Lactococcus lactis* FM03. Sustained growth at pH 8 triggers extensive physiological remodelling, including activation of general stress response proteins, suppression of acid resistance, elevated amino acid production, altered cell wall biosynthesis, and doubling of the cell wall thickness.

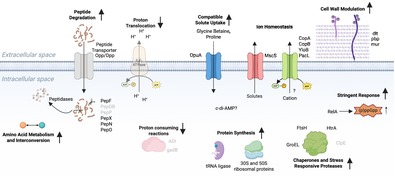

## 1 Introduction

*Lactococcus lactis* is a Gram‐positive lactic acid bacterium (LAB) extensively used in the food industry for its versatile fermentation capabilities. It is naturally adapted to both dairy and plant environments (Rademaker et al. 2007) and is even found in the gut of various insects (Choi et al. 2023). *Lactococcus* spp. plays a key role not only in flavour and texture development in plant‐ or dairy‐based products, but also ensures quality, safety and shelf‐life (Song et al. 2017). When applied as a starter culture or probiotic, *L. lactis* is exposed to a variety of stresses. For example, during starter culture production, cells are exposed to high osmolarity, freeze‐drying and extended storage (Ge et al. 2024). In the cheese‐making process, they must tolerate low pH, high osmolarity, nutrient starvation and heat (Sanders et al. 1999). Probiotic *L. lactis* cells face the human gastrointestinal tract, where they are exposed to bile and acid stress. Nevertheless, they must remain metabolically active and viable, rendering a stress response important to cope with continuous environmental changes. As their robustness as a starter culture is crucial for a successful product, their stress physiology, stress response mechanisms and stress tolerance strategies have been extensively studied (Wu et al. 2014; Bucka‐Kolendo and Sokołowska 2017; Sionek et al. 2024).

Although the majority of research has focused on acid stress, the stress response towards the other side of the pH scale—alkaline stress—has received little attention and is not well‐researched, apart from very few studies in lactobacilli (Sawatari and Yokota 2007; Lee et al. 2011; Wu et al. 2020) and *Enterococcus faecalis* (Acciarri et al. 2023, 2025). While non‐alkaliphilic LAB rarely encounter high pH in their natural niches, certain fermentation processes do subject LAB to alkaline conditions, where the pH is increasing due to the metabolic activity of other microbes present in their vicinity. For instance, *L. lactis* can experience alkaline pH in the rind of mould‐ripened cheeses. The rind can reach pH values above 8 due to the catabolism of amino acids by fungal microbiota consisting of multiple dairy yeasts and moulds such as *Geotrichum candidum* (Lessard et al. 2012; Unno et al. 2021). Also, for some fermentation processes, the initial pH is alkaline due to the pre‐treatment of the substrate, such as the lye‐treatment of green olives before fermentation with *Lactiplantibacillus pentosus* (Sánchez et al. 2001) or due to residues of cleaning detergents. Understanding the metabolism and stress response of *L. lactis* under alkaline conditions could help to ensure a stable fermentation process and optimised product quality under these conditions. Further, it will provide insights into the usability and stability of *L. lactis* in food products that undergo an alkaline treatment, to ensure a robust fermentation result, and it may open the doors for new product developments.

Known response mechanisms of non‐alkaliphilic LAB towards alkaline pH include the regulation of the intracellular pH by cation transport systems and proton‐translocating F_0_F_1_‐ATPases. Typically, lactococci have a flexible pH gradient (ΔpH) to adjust their cytoplasmic pH in response to the environmental conditions and maintain a near‐neutral cytosolic pH, critical for enzyme functionality and growth (Nannen and Hutkins 1991a; Molina‐Gutierrez et al. 2002). These systems are well studied in the context of acid stress, where they prevent cytoplasmic acidification by exporting protons, consuming protons intracellularly in decarboxylation reactions, or by producing NH_3_ via the arginine deiminase pathway. However, at neutral or alkaline conditions, the situation is fundamentally different. At neutral conditions compared with an acidic environment, ATPase activity in *L. lactis* is lower, resulting in a lower proton pumping capacity with increased pH conditions (Amachi et al. 1998). At pH values above 8, it is suspected that ΔpH is close to zero for non‐growing *L. lactis*, which would diminish the pH‐gradient component of the proton motive force (PMF) and render the PMF primarily dependent on the membrane potential (Δψ) (Kashket et al. 1980; Poolman, Smid, and Konings 1987; Konings 2002; Hansen et al. 2016). Moreover, an elevated intracellular pH might lead to conformational changes in proteins or impaired activities. To counter this effect, electrogenic K^+^/H^+^ (or Na^+^/H^+^) antiporters can be used to import protons in exchange for potassium (or sodium) efflux. This exchange system can contribute to maintaining the intracellular pH lower than the environmental pH and supports pH homeostasis in alkaline environments (Kashket et al. 1980). The same antiporters can also operate in the opposite direction under acidic conditions, exporting protons while importing K^+^ or Na^+^ to prevent excessive cytosolic acidification (Krulwich et al. 2009). However, accumulation of K^+^ and Na^+^ ions needs to be balanced to prevent osmotic stress and to maintain cytoplasmic homeostasis.

Despite the challenges *L. lactis* faces at alkaline pH, the alkaline environment may activate industrially relevant physiological responses, such as an enhanced robustness of starter cultures by cross‐resistance. It was observed that exposure to sublethal stresses can make *L. lactis* more resistant to the same stress or other stresses (Hartke et al. 1995, 1996; Wu et al. 2018). The cross‐protection is conferred due to some overlap in the induction of genes involved in stress defence (Hartke et al. 1996; Xie et al. 2004). For example, alkaline pH triggers the heat shock response in *E. coli* (Taglicht et al. 1987) and, thereby, induces chaperones. Elevated chaperone levels can be an interesting aspect of cheese making, as extended enzymatic activities contribute to a prolonged metabolic conversion and thus cheese ripening and flavour formation (Nugroho et al. 2021). Recent work in *Lactiplantibacillus plantarum* also indicated that pH 8 induces exopolysaccharide production and alters the expression of genes involved (Le et al. 2021; Nguyen et al. 2021).

Characterising the physiological changes and proteomic adaptations of *L. lactis* during exposure to alkaline conditions is the first step towards assessing the impact of alkaline pH and its industrial potential for application. In this context, the cheese isolate *L. lactis* subsp. *lactis* biovar. diacetylactis FM03 represents an interesting strain to study adaptations under alkaline conditions, due to its dairy origin, production of flavour compounds relevant for cheesemaking, robustness under stress conditions, and extensive plasmidome (van Mastrigt, Di Stefano, et al. 2018).

In this work, we characterised the alkaline tolerance response of *L. lactis* FM03. To study the long‐term physiological adaptations, *L. lactis* FM03 was habituated by prolonged chemostat cultivation at pH 6, 7, and 8, with pH 8 representing the alkaline condition. Through comparative proteomics and metabolomics, combined with morphological phenotyping, we identified underlying adaptation mechanisms that enable *L. lactis* to cope with alkaline environments. This approach provided new insights into how a non‐alkaliphilic LAB remodels its physiology to survive sustained growth at alkaline pH.

## 2 Materials and Methods

### 2.1 Culture Conditions and Chemostat Cultivation

The samples were harvested from a pH‐controlled chemostat with a fixed dilution rate of 0.2 h^−1^, with culture conditions as described in (Bendig et al. 2025). In short, *L. lactis* subsp. *lactis* biovar diacetylactis FM03 (van Mastrigt et al. 2017) was precultured overnight at 30°C in M17 (Difco, #218561) with 0.5% (w/v) lactose (LM17), before bioreactors (Multifors, Infors HT) were inoculated with 1% (v/v). The bioreactors had a working volume of 0.5 L and were run at 30°C under anaerobic conditions (0.1 L/min N_2_) with continuous pH control (5 M NaOH) and OD₆₀₀ monitoring (EXcell 231). The impeller speed was set to 200 rpm (300 rpm if signs of wall growth were observed, as 300 rpm reliably prevented visually detectable biofilm formation). Upon reaching the stationary phase, measured by a steady OD_600_, the continuous feeding of medium began at a fixed dilution rate of D = 0.2 h^−1^. Samples were taken after at least 5 volume changes from four biological replicates per pH and after stabilisation of biomass formation. The experiments were performed in filter‐sterilised (Minisart Syringe Filter, diameter 0.2 μm) chemically defined medium (CDM) (van Mastrigt, Mager, et al. 2018), supplemented with 0.24% (w/w) tri‐ammonium citrate, 1% tryptone (w/w) (Oxoid, LP0042) and 0.5% (w/w) lactose.

### 2.2 Analysis of Amino Acids

To measure amino acid concentrations, samples were collected from the chemostat, centrifuged (17,000 × g, 2 min, 4°C) and stored at −20°C until analysis. The free amino acids were measured with the AccQ‐Tag Ultra Derivatisation kit according to the manufacturer's instructions and as described by Scott Jr. et al. (2021). First, proteins were removed by adding 10 μL cold sulfosalicylic acid (0.3 g/mL) and 50 μL norvaline internal standard (250 μM norvaline in 0.1 M HCl) to 40 μL 1:1 diluted supernatant. After mixing, the sample was centrifuged at 17,000 × g for 10 min at 4°C, and the supernatant was collected. To derivatise the samples, 60 μL AccQ‐Tag Ultra Borate buffer (with 150 μL 4 M NaOH per 5 mL borate buffer) was added to a glass recovery vial with 20 μL supernatant of the protein‐removed sample. After mixing, 20 μL AccQ‐Tag Ultra derivation reagent was added, and immediately capped and thoroughly vortexed, followed by heating at 55°C for 10 min. The amino acids were quantified on an UltiMate 3000 (Dionex), equipped with an AccQ‐Tag Ultra BEH C18 column (Waters) and a BEH C18 guard column (Waters), with a column temperature of 55°C and a mobile phase flow rate of 0.7 mL/min. The mobile phase was 5% AccQ‐Tag Ultra concentrate solvent A and 100% AccQ‐Tag Ultra solvent B, with a separation gradient of following percentages of Eluent A: 0.00–0.04 min: 99.9%, 5.24 min: 90.9%, 7.24 min: 78.8%, 8.54 min: 57.8%, 8.55–10.14 min: 10%, 10.23–17.00 min: 99.9%.

### 2.3 Transmission Electron Microscopy

The thickness of the cell wall was quantified on images captured with transmission electron microscopy (TEM). The TEM images were only taken from one biological replicate per pH. To fixate the cells, 4 mL cell suspension harvested aseptically from the bioreactor was pelleted (17,000 × g, 2 min at 4°C), washed with peptone physiological salt (PPS; Tritium Microbiologie) and then resuspended in 2.5% glutaraldehyde in a 0.1 M phosphate/citrate buffer, pH 7.2. This suspension was kept at 4°C until analysis. Before microscopy, the supernatant was removed, and the sample was resuspended in 100 μL 3% gelatine in 0.1 M phosphate/citrate buffer, pH 7.2, for at least 20 min at 4°C. When the gelatine was solidified, the specimen was dried and cut into small pieces and incubated for at least 1 h in 2.5% glutaraldehyde in a 0.1 M phosphate/citrate buffer, pH 7.2, at room temperature, followed by six washing steps for 10 min each with 0.1 M phosphate/citrate buffer, pH 7.2. Then, the cells were stained with 1% osmium tetroxide in PPS for 1 h at room temperature and washed three times with 0.1 M phosphate buffer, pH 7.2, for 10 min each. The samples were dehydrated with a graded series of acetone (10%, 30%, 50%, 70%, 80%, 90%, 96%, 2 × 100%, 10 min each, and the last step 20 min), followed by infiltration with resin. The samples were transferred to BEEM (Spurr) and complexly filled with resin before being dried for 8 h at 70°C. The hardened samples were then sectioned using an ultramicrotome UC7 (Leica) and attached to 100 mesh carbon grids. The sample was incubated for 10 min in 2% uranyl acetate before being washed five times with MilliQ. Then, the grids were incubated for 10 min in a CO_2_‐free environment in lead citrate (EMS), washed two times in 0.01 N CO_2_‐free water and washed three times again with MilliQ. The images were captured with a JEM‐1400 microscope (JEOL) and an accelerating voltage of 120 kV.

The TEM images were analysed in ImageJ (V1.54p) (Schindelin et al. 2012). The scale was set automatically by detecting the pixel length of the scale bar with the corresponding nanometre value to define the scale in nm per pixel. To determine the cell wall thickness, we applied an ImageJ macro adapted from the Image.sc Forum (Patnaik 2020). Two lines marking the inner and the outer cell envelope were drawn manually, and the macro measured the shortest distance from 50 points between the two lines. Per pH condition, at least 20 cells with different magnifications were measured, with a resolution of 0.5 to 7.6 nm per pixel.

### 2.4 Proteomics and Statistical Analysis

Proteins were measured and quantified as described in Bendig et al. (2025). The proteomic samples were prepared in biological quadruplicates per pH condition, obtained from independent chemostat runs. Per sample, 2 mL cell suspension was pelleted (2 min, 17,000 × g, 4°C). The resulting pellet was washed twice with ice‐cold 100 mM Tris buffer, pH 8, with Pierce Protease Inhibitor (#A32965, Thermo Scientific), before resuspension and cell lysis by three rounds of sonication (15 s on and 30 s off) with a Soniprep 150 sonication probe (MSE) in the same buffer. The protein concentration was measured with the Pierce BCA Protein Assay (#23225, Thermo Scientific) according to the manufacturer's instructions. The proteins were reduced with 15 mM DTT, alkylated with 20 mM acrylamide and digested with sequencing‐grade trypsin (5 ng/μL) in 50 mM ammonium bicarbonate according to the protein aggregation capture (PAC) method (Batth et al. 2019). The resulting peptides were cleaned with an Empore C8 filter and reconstituted into 50 μL of 1 mL/L formic acid in water. The cleaned peptide samples were measured by injecting 5 μL into a Vanquish Neo nanoLC‐Orbitrap Exploris 480 MS/MS (Thermo Scientific) as described in Zheng et al. (2024). The MS data were analysed using the MaxQuant quantitative proteomics software package. False discovery rates were set to 0.01 for both protein and peptide levels. Database searches were performed with the Andromeda search engine against the proteome of *L. lactis* FM03 (Genome assembly ASM214821v1) and settings as described in Liu et al. (2021). The coding sequences of *L. lactis* FM03 of the genome and plasmids 1 to 12 are listed in Table S1. Digestion mode was set to “Specific Trypsin/P” with a maximum of two missed cleavages. The first‐search peptide tolerance was set to 20 ppm, the main‐search peptide tolerance to 4.5 ppm, and the MS/MS fragment match tolerance to 20 ppm. Propionamide (C) was set as fixed modification, while protein N‐terminal acetylation, methionine oxidation, and deamidation of asparagine and glutamine were set as variable modifications. The maximum number of modifications per peptide was set to five. The label‐free relative quantification was performed with the MaxLFQ algorithm to normalise the measured intensities. Only proteins identified with at least two peptides and that appeared in at least three out of the four biological replicates in at least 1 pH condition were used for relative quantification using LFQ intensities. Missing values were imputed from a narrowed (0.3 *σ*) and downshifted (1.8 *σ*) normal distribution by random sampling according to Lazar et al. (2016).

In this work, we focused mainly on changes in protein abundance reflected by their LFQ intensities between pH 6 and 8. A protein was considered differentially abundant if it had a one‐way ANOVA *p*‐value ≤ 0.05, an adjusted *p*‐value (Tukey HSD) for the comparison between pH 6 and pH 8 ≤ 0.05, and an absolute fold change between pH 6 and pH 8 of at least 1.5 (|log_2_ fold change| ≥ 0.585).

To identify groups with similar pH‐dependent abundance patterns, we performed a hierarchical clustering on the row‐wise scaled log_2_ intensity matrix of proteins with a significant (≤ 0.05) ANOVA *p*‐value, using the distance matrix computation implemented in R (dist(mat_scaled)) with Euclidean distance and complete linkage. Clusters were defined by cutting the resulting dendrogram at a fixed tree height (*h* = 1.4), yielding eight distinct protein clusters.

### 2.5 Enrichment Analysis

Functional enrichment analysis was conducted with FUNAGE (de Jong et al. 2022), using the set of proteins that were significantly up or downregulated (adjusted *p* ≤ 0.05 and absolute fold change ≥ 1.5). The resulting Gene Ontology (GO) terms were manually clustered into broader functional groups.

## 3 Results

### 3.1 Quality Control and Reproducibility

**FIGURE 1:**
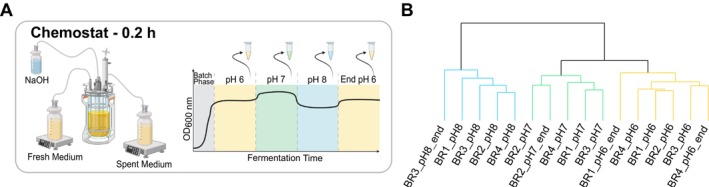
Schematic overview of the experimental setup and hierarchical clustering of samples. (A) Graphical representation of the chemostat setup. The order of the pH values per run was randomised. Per chemostat, samples were harvested at each pH, and the initial pH was reestablished as a control at the end of each cycle. Sampling was performed after at least five volume changes, corresponding to a minimum of 25 h at D = 0.2 h^−1^. (B) The hierarchical dendrogram represents the clustering of the proteomes by Wards' method from the Euclidean distance between the log_2_‐transformed LFQ intensities of the samples.

To study the proteomic adaptations to alkaline pH, we chose a pH‐controlled chemostat with a fixed dilution rate of 0.2 h^−1^. Each sample was harvested after the base addition rate and the optical density were constant, indicating a steady state, the earliest after five volume changes (Figure 1A). To rule out evolutionary effects, we randomised the order of pH values in each of the four chemostat runs and returned to the initial pH for the last sample. Clustering of the proteome samples revealed that each sample fell within one cluster per pH, including the end samples (Figure 1B). Beyond this clustering, the end samples were not included in the analysis. pH 6 and pH 7 were clustering closer together than pH 8, respectively, indicating a clear distinction in the proteome within each pH condition. The full clustering is shown in Figure S1. Volcano plots showed that the proteins separated most clearly between pH 6 and 8, and more weakly between pH 7 and 8, while there was little difference between pH 6 and 7 (Figure S2). We therefore focused mainly on the comparison between pH 6 and 8. Overall, we detected 1078 proteins in our samples, of which 457 were significantly differentially expressed with a *p*‐value below 0.05 according to ANOVA. Following a *post hoc* Tukey test, the majority, 379 proteins, were significantly changed between pH 6 and pH 8 (Figure S4). From those, 350 proteins showed at least 1.5‐fold up or downregulation at pH 8 compared with pH 6. One hundred seventy‐two proteins were at least 1.5‐fold upregulated and 178 proteins were at least 1.5‐fold downregulated. This indicates a significant change in the proteome upon alkaline conditions despite having the same growth rate.

### 3.2 Proteins With pH‐Dependent Expression Patterns

**FIGURE 2:**
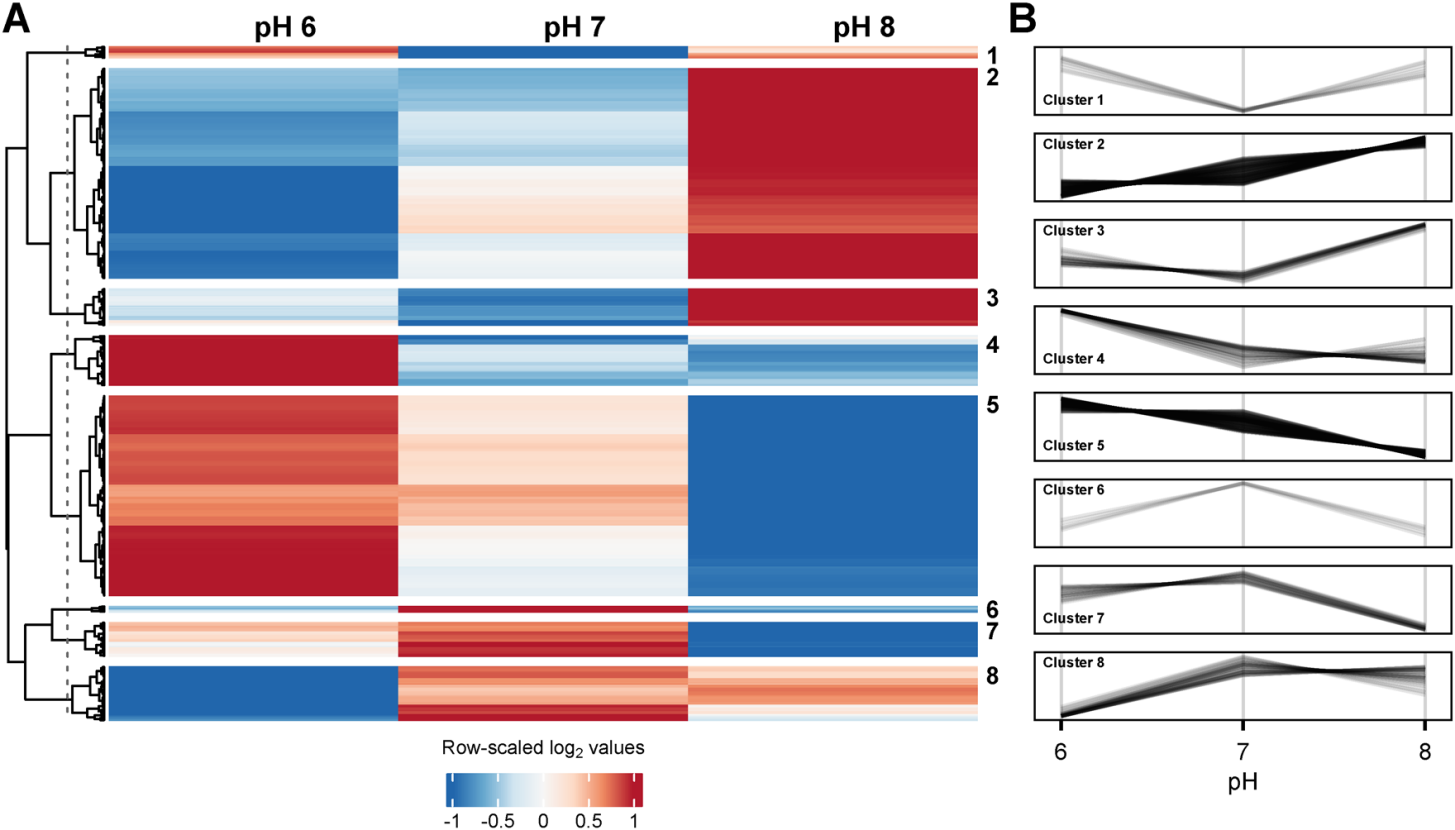
Hierarchical clustering of trend profiles in dependence on pH. (A) The log_2_‐transformed LFQ intensities of significantly differentially expressed proteins (ANOVA *p* ≤ 0.05) were scaled for each protein to identify clusters based on the trend profile. Splitting the dendrogram at a specific height (*h* = 1.4), indicated by the dashed line, revealed eight clusters. The resulting profiles of individual clusters are represented in (B). The profile of each protein was plotted with a slight transparency. Darker values represent a higher overlap.

To define the alkaline stress response of *L. lactis* FM03 and the pH‐dependent differences in the proteome, we clustered significantly differentially expressed proteins (ANOVA *p* ≤ 0.05) based on their abundance profiles. The log_2_‐transformed LFQ intensities were normalised, and hierarchical clustering was applied to group proteins with similar expression patterns (Figure 2). This revealed eight clusters: Cluster 2, 3, and 8 exhibited a higher abundance of the respective protein at pH 8 compared with pH 6, and cluster 4, 5, and 7 exhibited a lower abundance, respectively. Cluster 1 and cluster 6 show a V‐shaped pattern with an upregulation or downregulation at pH 6 and 8 relative to pH 7, respectively. Interestingly, the clusters with a gradual change in abundance (2 and 5) contained the most proteins.

To identify key adaptive mechanisms, we performed a functional enrichment analysis using differentially expressed protein clusters. Only proteins showing at least a 1.5‐fold change and an adjusted *p*‐value below 0.05 between pH 6 and 8 were included in the analysis, thereby excluding the V‐shaped clusters. Clusters 2, 3, and 8 (upregulated at pH 8) and clusters 4, 5, and 7 (downregulated at pH 8) were analysed using FUNAGE (de Jong et al. 2022) (Figure S3). Enrichment of Clusters of Orthologous Groups (COG) revealed a prominent overrepresentation of proteins involved in metabolic processes. In the upregulated clusters, we observed notable changes in pathways involving proteins related to translation (COG cluster J), amino acid transport (COG cluster E), and inorganic ion transport and metabolism (COG cluster P). This indicates changes in translation capacity, as well as an increased demand for amino acids and ions to cope with an alkaline environment. In contrast, the downregulated clusters were enriched for proteins involved in carbohydrate transport and metabolism (COG class G), energy production (COG class C), and coenzyme transport and metabolism (COG class H), suggesting metabolic adaptations such as the metabolic shift from mixed‐acid to homolactic fermentation as discussed in a previous work (Bendig et al. 2025). The group post‐translational modification, protein turnover, and chaperones (COG class O) was significantly enriched in the downregulated protein, but contained a similar number of proteins that were upregulated at pH 8.

Gene Ontology (GO) enrichment further supported these findings and defined adaptation mechanisms to alkaline conditions (Tables S3 and S4). Among the upregulated proteins, we identified significantly enriched pathways related to ion transport, compatible solute uptake, and the transport and metabolism of nucleotides and amino acids. Many of those transport processes were found in cluster 2 (gradually upregulated). Additionally, in cluster 2, we observed an enrichment of proteins involved in lipoteichoic acid (LTA) biosynthetic processes and penicillin‐binding activity, suggesting cell wall remodelling upon higher pH. tRNA ligases were also overrepresented among the upregulated proteins, hinting at broader adaptations in the translation machinery. Conversely, the downregulated proteins were primarily involved in carbohydrate metabolism, including phosphotransferase systems, glycogen synthesis, but also proton‐removing reactions such as arginine deiminase pathway, proton‐transporting ATP synthases, and citrate metabolism. Since citrate was present in the medium, the reduced citrate consumption rates with increasing pH, as discussed in Bendig et al. (2025), support the observed downregulation of citrate metabolism proteins.

In summary, the enrichment analysis revealed that *L. lactis* FM03 showed a diverse response to the elevated pH, including metabolic reprogramming, ion and solute transport, and adjustments in the cell wall and translation capacity. In the following sections, we will discuss the mechanisms identified from the enrichment analysis.

### 3.3 Alkaline pH Induces General Stress Responses and Inverts Acid Stress Mechanisms

**FIGURE 3:**
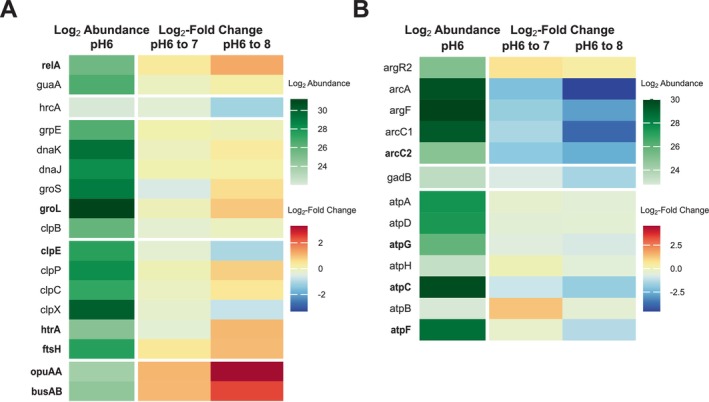
Stress response mechanisms altered by alkaline pH. Both heatmaps show the abundance of proteins at pH 6 (first column) and the log_2_‐fold change at pH 7 compared with pH 6 (second column) or 8 compared with pH 6 (third column). Higher protein abundance is indicated by darker shades of green. Red indicates higher abundance and blue lower abundance relative to pH 6. (A) General stress response mechanisms, including proteins of the stringent response, a regulator for chaperones, proteases, and transporters for the uptake of glycine betaine. (B) Common acid resistance mechanisms include proteins of the arginine deiminase pathway, the glutamate decarboxylase and subunits of the proton‐pumping F_0_F_1_‐type ATPases. Bold proteins are significantly up‐ or downregulated with at least a 1.5‐fold change in abundance and an adjusted *p*‐value ≤ 0.05 between pH 6 and pH 8. A complete overview of all fold‐changes and *p*‐values can be found in Table S2.

To assess whether *L. lactis* FM03 reacts with a stress response towards alkaline conditions, we analysed the abundance of proteins that are typically associated with general and acid stress adaptation (Figure 3). At pH 8, the GTP diphosphokinase RelA, which synthesises the stress alarmone (p)ppGpp, was significantly upregulated. Notably, the RelA expression increased between pH 6 and pH 8, and between pH 7 and pH 8, but not between pH 6 and pH 7, indicating that alkaline pH activates the stringent response.

Several chaperones and proteases involved in proteostasis, such as GroEL, GroES, HrcA, HtrA, and FtsH, had a higher abundance at higher pH, though not all changes were statistically significant. The upregulation of these chaperones and proteases was moderate and typically in the order of 2‐fold. However, it should be taken into account that DnaK and GroEL are highly abundant. For example, GroEL is among the 5% most abundant proteins, so a 2‐fold upregulation is already a large change in absolute terms. Interestingly, the negative regulator HrcA of the heat‐shock operons GrpE‐DnaK‐DnaJ and GroES, as well as GroEL, was 2.2‐fold downregulated (*p* = 0.052), suggesting a derepression of the heat‐shock regulon. However, the abundances of GrpE, DnaK and DnaJ remained stable across pH values. The expression patterns for proteases involved in protein recycling were variable. The protease ClpE, which is involved in the degradation of misfolded proteins (Ingmer et al. 1999), was two‐fold downregulated upon alkaline pH but not at pH 7. However, ClpP, the proteolytic subunit of the protein complex with ClpE, did not show a significant change.

As suggested by the GO‐term enrichment, alkaline pH upregulated transporters for compatible solutes. The abundance of the transporters for glycine betaine OpuAA (also named BusAA) and BusAB increased 9.9 and 5.4‐fold, respectively, at pH 8 compared with pH 6. Notably, both proteins increased gradually with increasing pH, despite the absence of glycine betaine in the medium.

Interestingly, acid stress response mechanisms were consistently and strongly downregulated at alkaline pH. Key pathways known for their upregulation at acidic pH in *L. lactis* , including the deiminase pathway (ADI), proton translocating F_0_F_1_‐ATPase, and the glutamate decarboxylase GadB, showed a coordinated repression at pH 8. Within the ADI pathway (ArcC1, ArcC2, ArgF and ArcA), the proteins were highly abundant at pH 6, but decreased at pH 7 (1.6 to 5.3‐fold) and even more at pH 8 (5.9 to 23.4‐fold). Taken together, these consistent decreases support the conclusion that acid stress systems are strongly repressed under alkaline conditions.

At pH 8, the GO‐term enrichment also suggested an increase in expression of proteins involved in cation transport. At pH 8, we observed an increase in the small mechanosensitive channel (MscS) and four P‐type cation ATPases (PacL1, YloB, CopA, CopB) (Figure S8). Phosphate and magnesium transport systems were unchanged. Sodium and potassium transporters were not detected in our proteome dataset, except for the potassium transporter KupB, which remained unchanged.

Taken together, these trends indicate a coordinated reprogramming of the stress proteome at pH 8, with the activation of the stringent response, a moderate activation of the general stress response, and a strong repression of acid stress mechanisms such as F_0_F_1_‐ATPases, the arginine deiminase pathway, and glutamate decarboxylation.

### 3.4 Cell Wall Thickening and Remodelling Upon Alkaline pH

**FIGURE 4:**
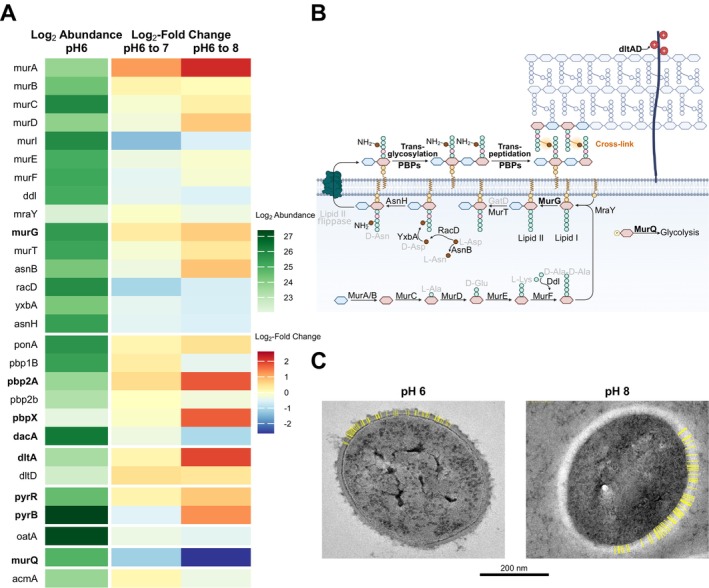
Peptidoglycan biosynthesis in *L. lactis* FM03 under alkaline conditions. (A) Heatmap of proteomic changes in key proteins involved in peptidoglycan synthesis, showing the abundance of proteins at pH 6 (first column) and the log_2_‐relative fold change at pH 6 to 7 (second column) or pH 6 to 8 (third column). Higher protein abundance is indicated by darker shades of green. Red indicates higher abundance and blue lower abundance relative to pH 6. Bold proteins are significantly up‐ or downregulated with at least a 1.5‐fold change in abundance and an adjusted *p*‐value ≤ 0.05 between pH 6 and pH 8. A complete overview of all fold‐changes and *p*‐values can be found in Table S2. (B) Schematic overview of peptidoglycan biosynthesis. UDP‐GlcNAc is shown in blue, UDP‐MurNAc in red. The pentapeptide stem is represented as green circles. Amidated D‐glutamate residues are highlighted in pink. The interpeptide bridge, formed by incorporation of D‐Asp or its amidated form D‐Asn, is shown in red. Lipoteichoic acids are drawn in dark blue, with D‐alanine ester modifications indicated as red positively charged circles. (C) Exemplary transmission electron micrographs of *L. lactis* FM03 cultivated in a chemostat with a dilution rate of 0.2 h^−1^ at pH 6 (left) or pH 8 (right). The yellow lines are measurement lines to determine the thickness. The thickness of the cell wall per cell was measured by averaging 50 lines.

To confirm the predicted changes in the cell wall structure, we quantified proteins associated with the peptidoglycan (PG) biosynthesis and measured the cell wall thickness using transmission electron microscopy (TEM). TEM images revealed an average cell wall thickness of 23.5 ± 2.4 nm at pH 6, 31.9 ± 7.3 nm at pH 7 and 44.3 ± 5.9 nm at pH 8. Proteomic analysis indicated that the abundances of proteins involved in the peptidoglycan biosynthetic machinery were significantly changed by pH (Figure 4).

Proteins of the first stage of the PG precursor synthesis (MurABCDEFGT and MraY) showed a *null* to weak but insignificant upregulation (Figure 4A). Only MurG, involved in the formation of lipid II, significantly increased 1.7‐fold. MurQ, the protein responsible for recycling N‐acetylmuramic acid (MurNAc) into glycolysis (Mayer et al. 2019), was strongly (6.1‐fold) downregulated at pH 8 as compared with pH 6, which might preserve MurNAc for reuse in the PG biosynthesis. After the PG precursor is flipped to the extracellular space, cross‐links between the PG subunits to an existing PG chain and between the pentapeptide chains can be formed by penicillin‐binding proteins (PBPs) (Courtin et al. 2006; Chapot‐Chartier and Kulakauskas 2014). Out of six PBPs found in *L. lactis* FM03, three were significantly changed. The high molecular weight Pbp2A (transglycosylase and transpeptidase) and PbpX (transpeptidase) were upregulated 3.4‐fold and 3.3‐fold, respectively. In contrast, the low molecular weight PBP DacA (D‐Ala‐D‐Ala‐carboxypeptidase) declined two‐fold. PBPs catalyse the cross‐linking between pentapeptide stems, which in *L. lactis* typically involve L‐Lys and D‐Asp (or D‐Asn) in the bridge. DacA trims the final D‐Ala residue, and its downregulation may preserve pentapeptides for cross‐linking (Courtin et al. 2006). Further, DacB (L‐, D‐carboxypeptidase), which further trims the pentapeptides to tripeptides, was 1.9‐fold downregulated. In addition, the abundance of AsnH, an asparagine synthase that amidates D‐Asp to D‐Asn in PG bridges (Veiga et al. 2009), was 1.5‐fold downregulated.

Apart from proteins directly involved with peptidoglycan synthesis, the D‐alanylation of lipoteichoic acids was also induced during growth at alkaline pH. D‐alanine is incorporated into the cell wall by the Dlt proteins (DltABCD) (Steen et al. 2005), which typically form a transcription unit. Among those proteins, the D‐alanine‐D‐alanyl carrier protein ligase DltA was 3.4‐fold upregulated upon a change in pH from 7 to 8 and 3.8‐fold from 6 to 8, while DltD was not affected by pH. DltB and DltC were not detected. Nevertheless, the strong upregulation of DltA may indicate a higher degree of D‐alanylation of LTA at alkaline pH.

### 3.5 High pH Enhances Amino Acid Metabolism and Proteolytic Activity

To determine how alkaline pH influences peptide utilisation and the amino acid metabolism, we analysed the abundance of proteins involved in peptide uptake, peptide degradation and amino acid metabolism, as well as the concentrations of free amino acids in the medium at a dilution rate of 0.2 h^−1^. The abundance of many of these proteins increased significantly, despite a small but insignificant upregulation in CodY (1.7‐fold, *p* = 0.06), the transcriptional repressor of several genes related to the proteolytic system and amino acid biosynthesis.

**FIGURE 5:**
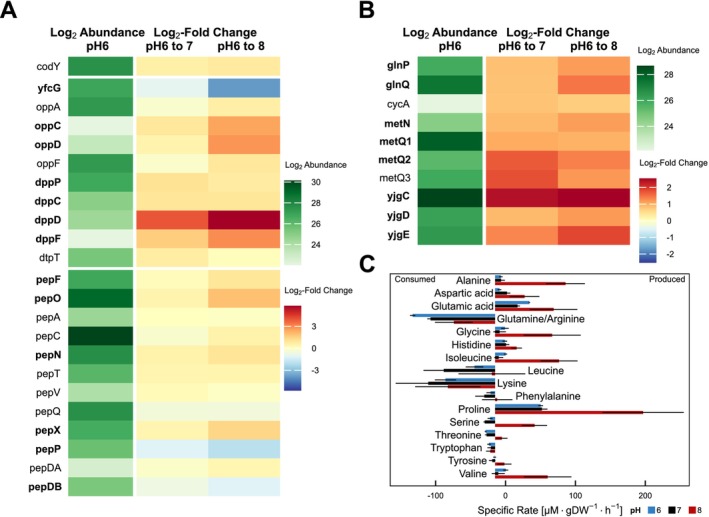
Amino acid metabolism and availability at different cultivation pHs. (A) and (B) Heatmaps showing proteins (A) related to peptide uptake, peptidases, and amino acid transferases and (B) involved in amino acid biosynthesis per amino acid. The first column represents the log_2_ abundance at pH 6, while the second and third columns represent the log_2_‐fold change between pH 6 and 7, and pH 6 and 8, respectively. Higher protein abundance is indicated by darker shades of green. Red indicates higher abundance and blue lower abundance relative to pH 6. Bold proteins are significantly up‐ or downregulated with at least a 1.5‐fold change in abundance and an adjusted *p*‐value ≤ 0.05 between pH 6 and pH 8. A complete overview of all fold‐changes and *p*‐values can be found in Table S2. (C) Biomass‐specific production rates of free amino acids in chemostat cultures in a CDM containing 1% peptide at a dilution rate of 0.2 h^−1^. The colours refer to the cultivation pH: pH 6 (blue), pH 7 (black), and pH 8 (red).

The proteolytic system of *L. lactis* FM03 lacks cell‐envelope proteinases, necessitating supplementation of the medium with peptides or free amino acids. In our case, we used a pancreatic digest of casein. Remarkably, many proteins belonging to the oligopeptide transporter as well as the di and tripeptides uptake systems, Opp and Dpp, respectively, showed a strong pH‐dependent gradual response (Figure 5A). While only OppCD increased significantly from the transport complex OppABCD, all found proteins (DppPCDF) for the Dpp complex increased significantly, with the strongest (more than 50‐fold) upregulation for the ATP‐binding protein DppD (Figure 5A). In contrast, the proton motive force‐driven di and tripeptide transporter DtpT did not show any pH‐dependent change. Additionally, transporters for individual amino acids, such as the glutamate/glutamine transporter GlnPQ, the methionine transporter MetN, and the amino acid permease YjgCDE, increased their abundance gradually with increasing pH, while the high‐affinity methionine uptake transporter MetQ (also referred to as PlpABC) increased at pH 7 and 8 compared with 6 (Figure 5B). The increase in relative abundance of transport systems of free amino acids, as well as peptides of various lengths, suggests either reduced transport efficiency at alkaline pH or an elevated demand for peptides and amino acids. In addition to an increased uptake, the abundance of 6 out of 12 found peptidases changed significantly in dependence on pH, most of them gradually with pH. The upregulation of endopeptidases PepF and PepO, the amino peptidases PepC and PepN and the proline peptidase PepX suggest a general activation of enzymes involved in the proteolytic cascade, to cleave more peptides into free amino acids. Conversely, the endopeptidase PepP, involved in protein maturation and the tri/dipeptidase PepDB are gradually downregulated, while the proline peptidase PepQ was only downregulated at pH 8.

Apart from proteins involved in peptide and amino acid transport, pathway analysis revealed that many enzymes involved in the biosynthesis or interconversion of amino acids were upregulated (Figures S7 and S8). For example, enzymes involved in the interconversion of glutamine and glutamic acid (glnA, and to a lesser extent pyrB, gltA, carB, carA), as well as the full biosynthesis pathway of serine and cysteine from 3‐phospho‐D‐glycerate (serABC, cysK1, cysK2), were markedly upregulated at pH 8.

Consistent with increased abundances of proteins involved in peptide uptake, hydrolysis and amino acid metabolism, the secretion rates of extracellular free amino acids increased at alkaline conditions (Figure 5C). Specific production rates per g dry weight were generally higher at pH 8 compared with pH 6 and 7. For threonine, serine, tyrosine, leucine and phenylalanine, we measured a positive specific rate at pH 8, while those amino acids were consumed at pH 6 and 7. Notably, we measured a higher L‐proline pool size in the supernatant, despite no proteomic indication of activated proline biosynthesis from glutamate, highlighting the importance of the peptidolytic system.

### 3.6 The Translational Apparatus Changes at Elevated pH

**FIGURE 6:**
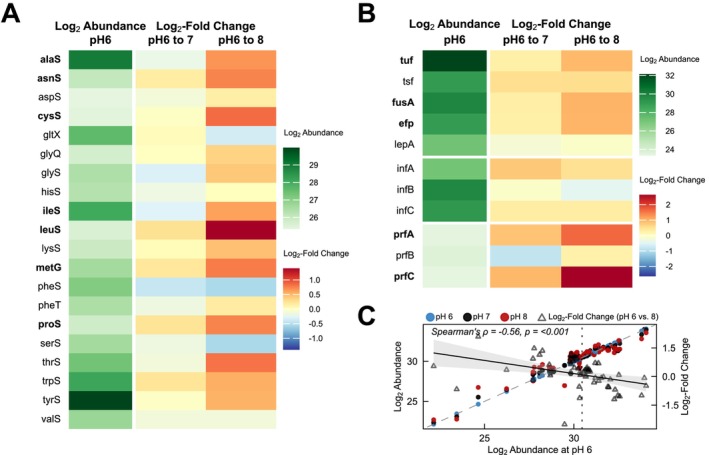
Proteins involved in translation as a function of cultivation pH. (A) and (B) Heatmaps showing the abundance of proteins for (A) Aminoacyl‐tRNA synthetases and (B) initiation, elongation, and termination factors at pH 6 (first column) and the log_2_‐fold change at pH 7 compared with pH 6 (second column) or 8 compared with pH 6 (third column). Higher protein abundance is indicated by darker shades of green. Red indicates higher abundance and blue lower abundance relative to pH 6. Bold proteins are significantly up‐ or downregulated with at least a 1.5‐fold change in abundance and an adjusted *p*‐value ≤ 0.05 between pH 6 and pH 8. A complete overview of all fold‐changes and *p*‐values can be found in Table S2. (C) Relative protein abundance (log_2_‐transformed) of 30S and 50S ribosomal proteins at pH 6 (blue), 7 (black), and 8 (red). The median for ribosomal proteins at pH 6 is shown with a vertical dotted line. The grey dashed line represents equal protein abundance at pH 6 (x = y). The black line shows the linear regression of fold changes between pH 6 and 8, as a function of protein abundance at pH 6. The grey shaded area denotes the 95% confidence interval of the regression fit.

The enrichment analysis further revealed adaptations in the translation machinery. We analysed the abundance of key components of the translation machinery, including ribosomal proteins and tRNA charging proteins. Interestingly, the 50S and 30S ribosomal proteins showed a converging trend towards similar abundances at alkaline pH, that is, higher expressed ribosomal proteins were often downregulated, while lower expressed ribosomal proteins were upregulated (Figure 6C), as indicated by a significant negative correlation (Spearman's *ρ* = −0.56, *p* < 0.001). Moreover, roughly half of the aminoacyl‐tRNA ligases increased at pH 8, with an average 1.6‐fold change (Figure 6A). Only the serine tRNA ligase (serS) showed a 1.5‐fold lower downregulation. Several translation factors were upregulated (Figure 6B) and initiation factors (InfABC) showed a stable expression. In contrast, the elongation factor EF‐Tu (tuf), EF‐G (fusA), EF‐P (efp) and termination factors (PrfAC) were upregulated at higher pH. These findings suggest an overall alteration in the translational machinery at alkaline pH.

## 4 Discussion

Lactococci are best known for thriving in mildly acidic environments, yet they have demonstrated a remarkable resilience towards a variety of environmental stresses, including temperature shifts, hyperosmolarity and acidic pH. This stress tolerance raises the question of how far their physiological flexibility and adaptability extend when they are exposed to alkaline pH, a condition outside their typical growth range and ecological niche. While the acid stress response is well researched, comparatively little is known about the alkaline stress response in non‐alkaliphilic LAB. This leads to a central question: how does a non‐alkaliphilic microbe like *L. lactis* deal with an unfamiliar high pH? To address this, we characterised the alkaline response of *L. lactis* FM03 grown in a chemostat with a fixed dilution rate of 0.2 h^−1^, thereby habituating the cells to the respective pH condition. It was shown that microbial stress responses can differ depending on whether cells experience stress by gradual exposure or habituation (Papadimitriou et al. 2008). Our experimental design specifically targeted mechanisms employed during sustained and prolonged growth under alkaline conditions, as could be found, for instance, in the rind of mould cheeses where the pH exceeds 8.

Our proteomic analysis revealed a substantial number of significantly up or downregulated proteins and a plethora of enriched GO terms. Those proteomic abundance changes, together with experimental measurements of the extracellular amino acid pools and cell wall thickness, as well as literature evidence, provide insights into potential adaptation mechanisms employed by *L. lactis* to cope with alkaline stress.

### 4.1 Global Stress Response

While an environmental pH of 8 is rather mildly alkaline, our proteomic data suggest that *L. lactis* FM03 perceives pH 8 as a stressful environment. The physiological burden of the alkaline pH is reflected by a lower cell density (Bendig et al. 2025) and extensive proteome remodelling (this work), including the activation of proteins commonly induced in response to stress, such as heat, salt or acidic pH (Xie et al. 2004). As *L. lactis* does not possess alternative sigma factors such as *σ* ^B^ or *σ* ^S^ to orchestrate a general stress response (van der Meulen et al. 2016), it relies on other regulatory mechanisms. One such regulator is the stringent response, triggered by an increasing intracellular pool of the alarmone (p)ppGpp (guanosine tetraphosphate or pentaphosphate). In an environment perceived as stressful, this alarmone is helping the cell to switch from a growing state to a survival mode by triggering a cascade of adaptive responses. The upregulation of the primary (p)ppGpp synthetase RelA at pH 8 compared with pH 6 and 7 may lead to this accumulation of (p)ppGpp (Figure 3A). However, direct measurement of intracellular (p)ppGpp levels would be required to confirm the activation of the stringent response in *L. lactis* FM03 at pH 8. Nevertheless, elevated pH has previously been shown to cause ppGpp accumulation in *Staphylococcus aureus* (alkaline shock at pH 10, Anderson et al. 2010) and in *Pseudomonas aeruginosa* (grown at pH 8.5, Boes et al. 2008). In our data, the potential activation of (p)ppGpp signalling is consistent with enhanced amino acid biosynthesis and uptake (discussed below). The abundances of the regulators CcpA, involved in carbon catabolite control, and CodY, involved in nitrogen metabolism, remained unchanged upon alkaline pH, suggesting that the stringent response was not triggered by nutrient starvation.

Stress‐related proteins showed a selective response (Figure 3A). The abundance of FtsH, a membrane‐associated metalloprotease responsible for protein quality control and degrading cytoplasmic as well as membrane proteins (Nilsson et al. 1994), gradually increased with rising pH, together with the abundances of the surface‐localised protease HtrA (Poquet et al. 2000) and the chaperonin GroEL. GroES, the partner of GroEL, also trended upwards (1.5‐fold, *p* = *0.07*). In contrast, neither the molecular chaperones DnaJ and DnaK increased, nor did the nucleotide exchange factor Grp needed for the activity of DnaK. Notably, the protease ClpE was found to be gradually downregulated. Together, this indicates a tailored proteostasis response, with an increase in membrane protein quality control and increased reliance on GroEL, rather than a broad induction of stress proteins. The lack of a general upregulation of all stress proteins upon growth at alkaline pH might be due to the prolonged exposure, in contrast to studies that focus on alkaline shock in other microorganisms (e.g., Flahaut et al. 1997).

### 4.2 pH Homeostasis and Acid Stress Response

Our proteome dataset indicates a strong downregulation of important acid stress resistance systems during growth at alkaline pH (Figure 3B), including the ATP‐driven proton efflux pump F_0_F_1_‐ATPases (O'Sullivan and Condon 1999), the arginine deiminase pathway (Marquis et al. 1987; Cotter and Hill 2003), and the glutamate decarboxylase system (Sanders et al. 1998). Under acidic conditions, those three mechanisms remove cytosolic protons, thereby supporting intracellular pH homeostasis and ΔpH. In fermentative lactococci such as *L. lactis*, the F_0_F_1_‐ATPase mainly functions as an ATP‐consuming proton pump under physiological conditions, exporting protons to produce ΔpH (and to a lesser extent ΔΨ) (Kakinuma 1998). However, at alkaline pH, ΔpH decreases strongly and may approach zero (Kashket et al. 1980; Poolman, Hellingwerf, and Konings 1987; Konings 2002; Hansen et al. 2016), limiting proton‐coupled transport processes. Therefore, the downregulation of F_0_F_1_‐ATPase subunits at pH 8 is consistent with the reduced reliance on ATP‐driven proton extrusion and potentially lower proton‐pumping capacity. Those results are also in line with prior reports that the F_0_F_1_‐ATPase activity declines rapidly above pH 7.5 (Nannen and Hutkins 1991b) and with a lower need to export protons when the extracellular pH is high (O'Sullivan and Condon 1999; Frees et al. 2003). Similarly, the lower‐abundant arginine deiminase and glutamate decarboxylase systems at pH 8 may reflect the lower need for proton‐consuming reactions due to a lower cytosolic proton availability. Repressing those pathways may reduce unnecessary energy investments under alkaline conditions, when intracellular protons are scarce and excessive loss or consumption of cytosolic protons would alkalise the cytosol. Further studies on the intracellular pH, PMF and intracellular ATP levels would provide interesting insights into the bioenergetics and ATP demand under alkaline conditions.

### 4.3 Ion Homeostasis and Turgor Under Alkaline Conditions

Growth at alkaline pH comes with an energetic challenge for *L. lactis* , as ΔpH diminishes towards zero or can even reverse (i.e., the cytosol becomes more acidic) as the environmental pH approaches 8 (Poolman, Driessen, and Konings 1987; Breeuwer et al. 1996; Molina‐Gutierrez et al. 2002; Hansen et al. 2016). Under these conditions, the PMF becomes mainly dependent on ΔΨ (Kashket et al. 1980; Poolman, Smid, and Konings 1987). At the same time, *L. lactis* needs to maintain turgor and therefore typically contains high levels of K^+^. Since K^+^ uptake is electrogenic, excessive influx leads to a net entry of positive charges, thereby depolarising ΔΨ (Kashket and Barker 1977; Kashket et al. 1980). This creates a conflict between maintaining turgor and preserving ΔΨ at alkaline pH.

One possible strategy to reduce this conflict is by accumulating counter‐anions (mainly glutamate) and/or pools of electroneutral compatible solutes (e.g., glycine betaine or proline), that replace K^+^ as osmolytes without adding charge. Typically, glycine betaine is used to balance osmotic stress (Csonka 1989; van Der Heide and Poolman 2000). However, glycine betaine was shown to promote growth at pH 8.2, while simultaneously lowering intracellular K^+^ pools in *Escherichia coli* (Smirnova and Oktyabrsky 1995). This indicates that accumulating glycine betaine might help to alleviate alkaline stress by substituting K^+^. Other advantages of glycine betaine are its high‐affinity ATP‐driven uptake, allowing for large intracellular pools and its role in stabilising enzyme activities under unfavourable conditions (Lippert and Galinski 1992; Singh et al. 2009). Considering its natural presence in goat and cow milk (Zivkovic et al. 2021), future studies aiming to investigate the role of glycine betaine in alkaline adaptation and its potential benefits for dairy‐associated strains could yield insightful results.

In our chemostat cultures, the glycine betaine transporter OpuA (OpuAA [also called BusAA] and BusAB) was strongly upregulated (Figure 3A), although our medium lacked glycine betaine. Even in the absence of glycine betaine, expression of OpuA can increase, as its expression depends on the nucleotide second messenger cyclic‐di‐AMP (c‐di‐AMP) (van Der Heide and Poolman 2000; Sikkema et al. 2020). C‐di‐AMP is the master regulator for osmoresistance, turgor, and cell volume in Gram‐positive bacteria and controls, among others, the intracellular pools of K^+^ and compatible solutes (Pham et al. 2018; Turner et al. 2023; Foster et al. 2024). C‐di‐AMP can bind to the C‐terminal RCK_C domain of BusR, thereby repressing the genes *opuAA* (also called *busAA*) and *busAB*. In addition, c‐di‐AMP can inhibit K^+^ uptake by binding the high‐affinity K^+^ importer KupB as shown in *L. lactis* IL1403 (Quintana et al. 2019).

The high abundance of OpuA at pH 8 in our data may reflect low c‐di‐AMP levels, which may increase K^+^ uptake through KupB, potentially contributing to pH homeostasis via K^+^/H^+^ antiporters. While KupB abundances did not change with variations in pH, proteomics does not capture regulations through ligand binding or other posttranslational mechanisms. Low c‐di‐AMP levels would also align with enlarged cells at pH 8 (Bendig et al. 2025), suggesting a higher turgor and the increased cell wall thickness (Figure 4C), similar to effects observed in *Lb. plantarum* (Yu et al. 2025). The possible link between alkaline pH, altered c‐di‐AMP levels, K^+^ homeostasis, compatible solute accumulation and cell morphology remains hypothetical and requires experimental quantification under different pH conditions.

While K^+^/H^+^ and Na^+^/H^+^ antiporters and symporters have a vital role in alkaline pH homeostasis, we did not detect such transporters in our proteome dataset (Padan et al. 2005; Nyanga‐Koumou et al. 2012). Their absence might be due to low abundance and/or loss in the extraction procedures. The genome of *L. lactis* FM03 encodes the high‐affinity K^+^ uptake system KupA and KupB, and two putative NhaP/NapA‐type Na^+^/H^+^ (K^+^/H^+^) antiporters, whereas Ktr/Trk systems were absent. However, several cation‐translocating P‐type ATPases (CopA, CopB, YloB, PacL1) and the small conductance mechanosensitive channel MscS were significantly upregulated (Figure S6). The currently known function of those P‐type ATPases is primarily for metal homeostasis (Odermatt et al. 1994; Magnani et al. 2008), whereas MscS opens under membrane tension to rapidly release cytosolic osmolytes, serving thereby as a fast “safety valve” to reduce turgor pressure (Folgering et al. 2005; Booth and Blount 2012). Alkaliphiles such as *Enterococcus hirae* rely on V_0_V_1_‐type Na^+^‐translocating ATPases for alkaline adaptations, when the proton‐dependent systems become less effective due to a collapsing pH gradient (Kakinuma 1998; Kawano et al. 1998). While the four P‐type cation ATPases are not homologues of the Na^+^‐ATPases of *E. hirae* , their altered protein abundancies and their potential role under alkaline pH‐homoeostasis makes them interesting targets for future studies.

### 4.4 Translational Machinery and Quality Control

RNA is more susceptible to alkaline pH than DNA, as RNA is prone to base‐catalysed hydrolysis at pH values above 6, whereas DNA remains stable up to pH 10 (Oivanen et al. 1998; Bivehed et al. 2023). In line with this, we observed little effect on proteins involved in DNA repair (Figure S5). In contrast, aminoacyl‐tRNAs are destabilised by the accelerated alkaline hydrolysis of the phosphate anhydride bonds under alkaline conditions (Schuber and Pinck 1974). This may explain the upregulation of many tRNA ligases and the elongation factor EF‐Tu, which shields the aminoacyl bond from hydrolysis (Figure 6B) (Bernhardt and Tate 2012; Peacock et al. 2014). 30S and 50S ribosomal proteins also showed a rebalancing pattern: low‐expressed proteins tended to increase, while high‐abundant ribosomal proteins tended to decrease, converging towards a similar level (Figure 6C). Similarly, elongation and termination factors have been upregulated. Together, these adaptations may help to buffer the destabilising effect of alkaline pH and secure translation efficiency despite more fragile aminoacyl‐tRNAs.

### 4.5 Peptide and Amino Acid Metabolism

A striking adaptation was the strong upregulation of peptide transporters and amino acid metabolism at higher pH. It is known that glutamate is growth‐limiting at alkaline pH in *L. lactis* due to transport constraints, as only the protonated species (glutamic acid) can be transported by GlnPQ (Poolman and Konings 1988). Our proteome dataset suggests that this may be bypassed by the upregulation of components of the peptide uptake systems (Opp and Dpp), several peptidases, as well as amino acid biosynthetic pathways and amino acid interconversion (Figure 5A,B, Figures S7 and S8). As *L. lactis* FM03 is non‐proteolytic, we added tryptone, the pancreatic digest of casein, to the medium used in our chemostat fermentations. This casein digest contains peptides of various lengths, as well as free amino acids, thereby targeting different peptide and amino acid uptake systems. The relative abundance of the Dpp transport system was gradually increasing with higher pH values, while the DtpT system remained unaffected, which is logical considering their uptake mechanisms. The DtpT system relies on the proton motive force, and peptides are taken up in symport with a proton, while the Dpp system is ATP‐dependent (Smid et al. 1989; Hagting et al. 1994). This renders the ATP‐driven Dpp system more effective under alkaline conditions. The combination of upregulated transport as well as intracellular peptidases might create amino acid levels that exceed metabolic demand, which is reflected by the higher production rates of amino acids in the supernatant (Figure 5C). Several amino acids (histidine, serine and asparagine) can become growth‐inhibiting when their cytosolic concentrations rise too high (Meißner et al. 2022; Warneke et al. 2024). The upregulation of the mechanosensitive channel MscS may indicate altered membrane tension or turgor homeostasis, potentially also due to increased intracellular amino acid levels, and could contribute to the release of small cytosolic solutes. Nevertheless, an increased amino acid secretion has industrial relevance, as amino acids are precursors of flavour components in dairy products like cheese (van Kranenburg et al. 2002).

### 4.6 Cell Wall

A sturdy cell wall can offer protection towards a harsh environment. When *L. lactis* was grown at alkaline conditions, we observed an approximately two‐fold thicker peptidoglycan (PG) layer, accompanied by changes in proteins involved in PG biosynthesis, cross‐linking, and LTA decoration (Figure 4). Cell wall plasticity is the result of a balance between multiple processes to ensure rigidity, yet flexibility. While the cell wall needs to be flexible in growing cells, it can be more advantageous to be more rigid in the stationary phase.

The plasticity of the cell wall depends on the balance between synthesis, turnover, cross‐linking and surface modifications. While the core assembly of the PG subunits remained largely unaffected by pH, the strong downregulation of the N‐acetylmuramic acid 6‐phosphate etherase MurQ suggested a shift in flux of MurNAc from salvage and glycolysis towards PG synthesis (Figure 4A). This potentially favours structural reinforcement over salvaging (Mayer et al. 2019). Measuring the cell wall composition or fluxes of PG subunits in future studies could provide valuable insights into reinforcements under alkaline conditions.

The abundance of several enzymes involved in PG cross‐linking was modulated by pH. Upregulation of Pbp2A and PbpX, along with downregulation of DacA, may increase the proportion of pentapeptide stems available for cross‐linking (Figure 4A). Increased cross‐linking has previously been associated with higher cell wall rigidity (Roces et al. 2012; Loskill et al. 2014), while reduced levels of the D‐alanyl‐D‐alanine carboxypeptidase may thicken the cell wall, as observed in *Listeria monocytogenes* strains lacking D‐alanyl‐D‐alanine carboxypeptidase (DacA in *Lactococcus* spp., (Korsak et al. 2005)). Cross‐linking can also be influenced by amino acid availability. At pH 8, extracellular L‐aspartate concentrations increased, which could increase the substrate pool for D‐aspartate formation and incorporation into peptide stems by RacD and YxbA (also referred to as AslA). Despite PyrB upregulation, which may divert aspartate towards pyrimidine biosynthesis and stable levels of RacD and YxbA, the higher availability of D‐aspartate at pH 8 may cause its higher incorporation into PG, thereby facilitating cross‐linking. However, the slight (1.5‐fold) downregulation of AsnH implies reduced amidation of D‐aspartate, and thus potentially less D‐asparagine in the interpeptide bridge. Reduced amidation was shown to affect autolysis rate and lysozyme resistance (Veiga et al. 2009; Hao et al. 2017), and a higher amidation is utilised under acid stress to enhance acid resistance (Hao et al. 2017). Its downregulation at alkaline pH appears to be the opposite strategy.

In parallel, DltA was more abundant at pH 8, pointing towards a possible increased D‐alanylation of LTAs. This modification decreases the net anionic charge of the phosphate groups, which can improve the resistance to cationic antimicrobials and, in some cases, phage resistance in Gram‐positive bacteria (DltA and DltD) (Fallico et al. 2011). Deletion of any of the Dlt proteins or the depletion of D‐alanine increased cell lysis (Wecke et al. 1996; Steen et al. 2005). Similarly, in *L. lactis* F44, an increase in Dlt proteins enhances the acid tolerance (Wu et al. 2022). The increased availability of L‐alanine in the medium (Figure 5), together with increased levels of DltA (Figure 4A), may suggest a boosted LTA D‐alanylation. However, the half‐life of D‐alanyl‐LTA is severely reduced by increased pH, as the ester linkage is labile under alkaline pH (Childs and Neuhaus 1980). Thus, the increased DltA abundance may only indicate an altered or compensatory D‐alanylation capacity rather than an actual increase in LTA D‐alanylation. Measurements of this modification would be required to determine the impact of pH on the actual cell envelope charge.

## 5 Conclusion

**FIGURE 7:**
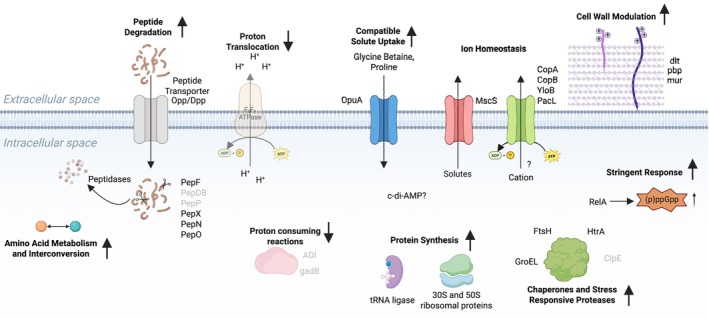
Graphical representation of alkaline resistance mechanisms in alkaline pH‐habituated *L. lactis* FM03 discussed in this work. The mechanisms were derived from the proteomic data of four independent chemostats at pH 6, 7 and 8. Upregulated processes are in black, and downregulated processes are shown in semi‐transparent grey.

Taken together, our proteomic dataset revealed that *L. lactis* FM03 showed a diverse response to alkaline stress with a coordinated adjustment of its physiology (Figure 7). While alkaliphiles typically boost Na^+^ or K^+^ transport for pH homeostasis, we only detected one such transporter, KupB, whose abundance was not altered in the proteome dataset. However, the upregulation of the glycine betaine transporter OpuA suggested low c‐di‐AMP levels, which also favours the uptake of K^+^ by the high‐affinity transporter KupB. Further, *L. lactis* shut down acid stress resistance mechanisms that consume or extrude protons. In parallel, the cell wall was reinforced through altered cross‐linking, amidation, and D‐alanylation, resulting in a doubling of the cell wall thickness. The translational machinery was remodelled to counteract alkaline‐driven RNA instability, and amino acid uptake was reprogrammed towards ATP‐dependent instead of PMF‐dependent transport systems. Peptide transporters and peptidases, as well as proteins in amino acid metabolism, were increased, reflected by higher extracellular amino acid production rates. Together, these findings reveal that *L. lactis* FM03 deals with an unfamiliar high pH through a coordinated physiological rewiring, with metabolic adjustments and cell envelope remodelling to sustain growth under alkaline stress.

## Author Contributions

**Tjakko Abee:** writing – review and editing, funding acquisition, supervision. **Tamara A. L. Bendig:** writing – review and editing, writing – original draft, investigation, methodology, conceptualization, software, data curation, formal analysis, validation, visualization. **Oscar van Mastrigt:** writing – review and editing, funding acquisition, supervision, project administration, conceptualization. **Eddy J. Smid:** supervision, project administration, writing – review and editing, conceptualization, funding acquisition, resources. **Sjef Boeren:** formal analysis, software, methodology.

## Funding

This work was supported by Nederlandse Organisatie voor Wetenschappelijk Onderzoek, 18048.

## Conflicts of Interest

The authors declare no conflicts of interest.

## Supporting information


**Figure S1:** Clustering and dendrogram of all quantified proteins across the individual proteome samples.
**Figure S2:** Volcano Plot of pairwise proteome comparisons between pH conditions.
**Figure S3:** COG Enrichment analysis of proteins differing between pH 6 and pH 8.
**Figure S4:** Proteome coverage and differential protein abundance overview for *L. lactis* FM03.
**Figure S5:** DNA repair mechanisms.
**Figure S6:** Cation transporters.
**Figure S7:** Amino acid biosynthesis and interconversion pathways affected by alkaline pH in *L. lactis* FM03.
**Figure S8:** Amino acid biosynthesis and interconversion pathways of glutamine, arginine and tyrosine in *L. lactis* FM03 affected by alkaline pH.

## Data Availability

The data that support the findings of this study are openly available in the PRIDE partner repository at www.ebi.ac.uk/pride, reference number PXD065893.
