# Supplementary material for: Proteomic Analysis of Lactococcus lactis FM03 Under Prolonged Alkaline Stress
Source: Environ Microbiol. 2026 Jul 17;28(7):e70381. doi: 10.1111/1462-2920.70381 (PMC13379511; doi:10.1111/1462-2920.70381)
Supplement: Supplementary file 1 — Figure S1: Clustering and dendrogram of all quantified proteins across the individual proteome samples. Figure S2: Volcano Plot of pairwise proteome comparisons between pH conditions. Figure S3: COG Enrichment analysis of proteins differing between pH 6 and pH 8. Figure S4: Proteome coverage and differential protein abundance overview for L. lactis FM03. Figure S5: DNA repair mechanisms. Figure S6: Cation transporters. Figure S7: Amino acid biosynthesis and interconversion pathways affected by alkaline pH in L. lactis FM03. Figure S8: Amino acid biosynthesis and interconversion pathways of glutamine, arginine and tyrosine in L. lactis FM03 affected by alkaline pH. [file EMI-28-e70381-s001.pdf]

Proteomic analysis of *Lactococcus lactis* FM03 under prolonged alkaline stress

Tamara Bendig[a], Tjakko Abee[a], Eddy J. Smid [a], Sjef Boeren[b], Oscar van Mastrigt[a*]

[a] Food Microbiology, Wageningen University and Research, Wageningen, The Netherlands

[b] Laboratory of Biochemistry, Wageningen University and Research, Wageningen, The Netherlands

Keywords: high pH, proteomics, physiology, lactic acid bacteria, stress response

***Corresponding Author**, Oscar van Mastrigt, oscar.vanmastrigt@wur.nl, Bornse Weilanden 9, 6708 WG Wageningen, The Netherlands

Supplement

Proteomic Data

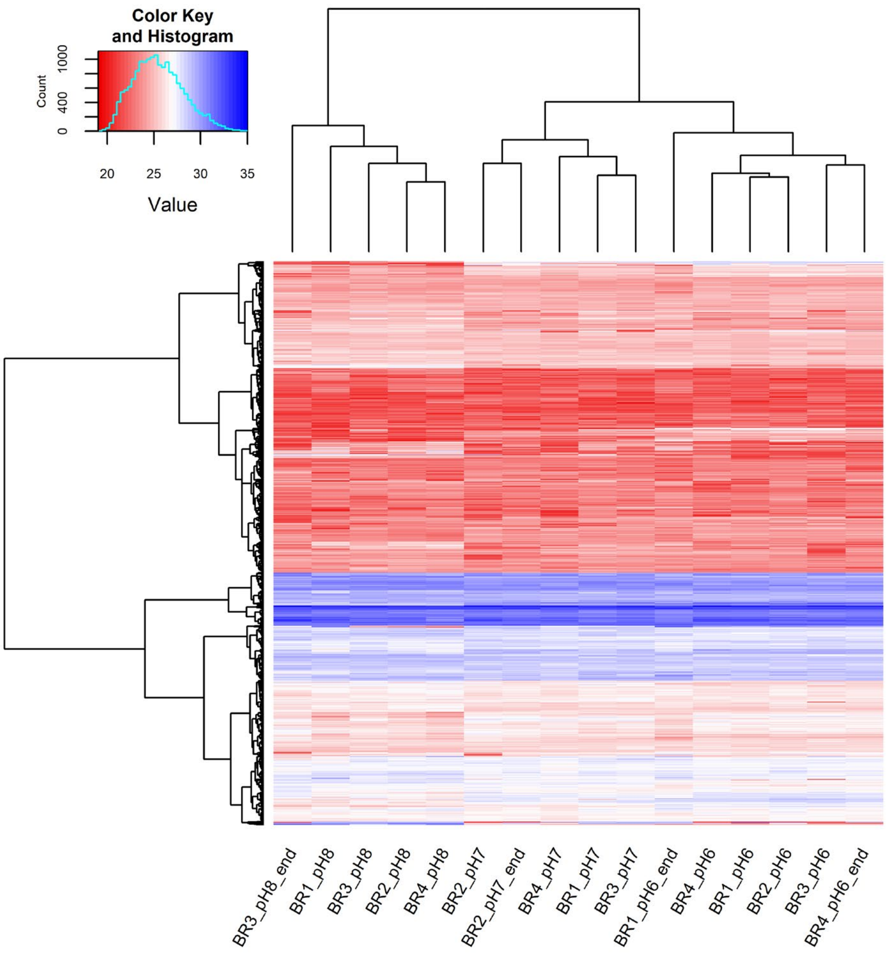

Supplement Figure S1: Clustering and dendrogram of all quantified proteins across the individual proteome samples. The hierarchical dendrogram represents the clustering of the proteomes by Wards' method from the Euclidean distance between the log_2-transformed LFQ intensities of the samples. Rows represent proteins, and columns represent the samples of independent biological replicates. The colour scale indicates the relative LFQ intensity value, with blue representing a higher abundance and red a lower abundance. This full clustering corresponds to the compact overview shown in Figure 1B.

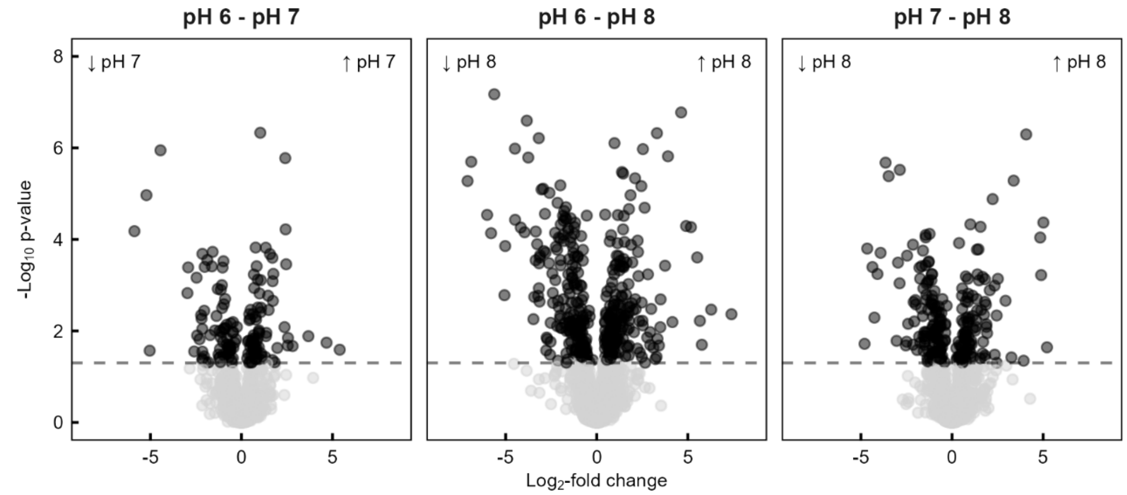

Supplement Figure S2: Volcano Plot of pairwise proteome comparisons between pH conditions. Each point represents one quantified protein with the log2-fold change, with proteins enriched at the lower pH on the left side. The *p*-value was calculated from imputed log_2-transformed LFQ data using ANOVA and *post hoc* Tukey. The $-log_{10}$-transformed *p*-value from Tukey was used to generate the plot. The dashed horizontal line indicates the significance threshold of $p \geq 0.05$.

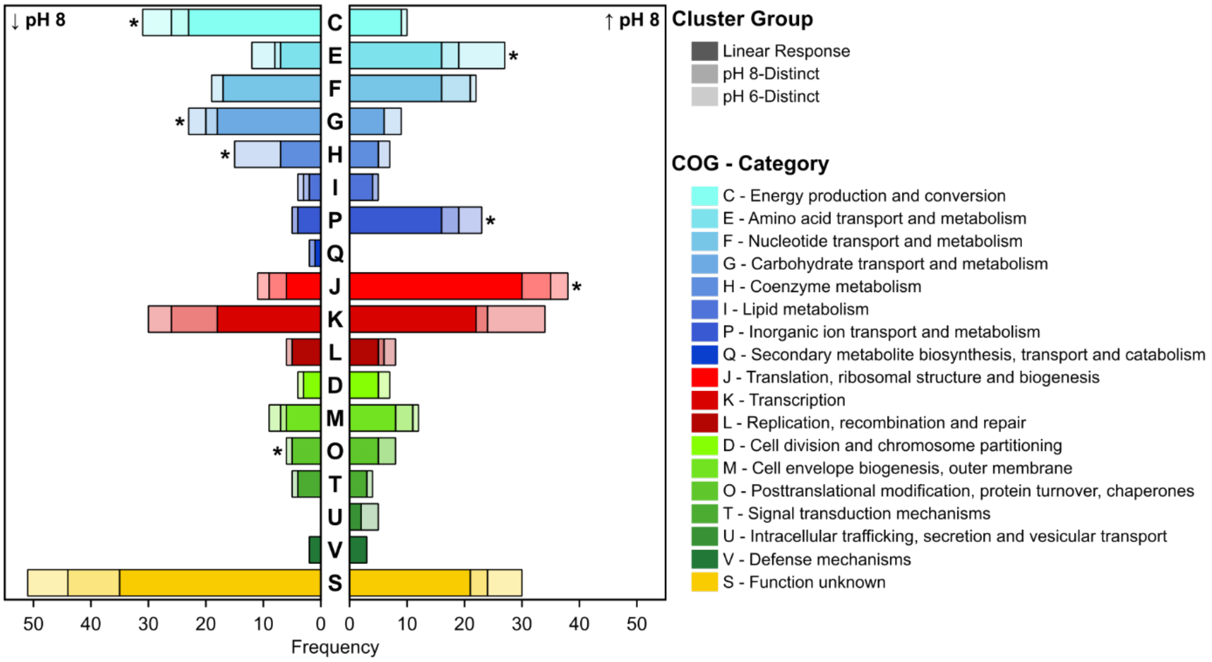

Supplement Figure S3: COG Enrichment analysis of proteins differing between pH 6 and pH 8. The functional classification of all proteins was carried out using the tool eggNOG-mapper v2 (Cantalapiedra et al., 2021) with the database EggNOG v5.0 (Huerta-Cepas et al., 2018), based on the coding sequences of *L. lactis* FM03 chromosome and plasmids 1 to 12 (Supplement Table S1). Further analysis was performed by categorising the proteins into Clusters of Orthologous Groups (COGs), with an enrichment analysis using FUNAGE (de Jong et al., 2022). Significantly enriched ($p \leq 0.05$) groups are indicated with an asterisk. COG categories which are upregulated at pH 8 compared to pH 6 are shown on the right.

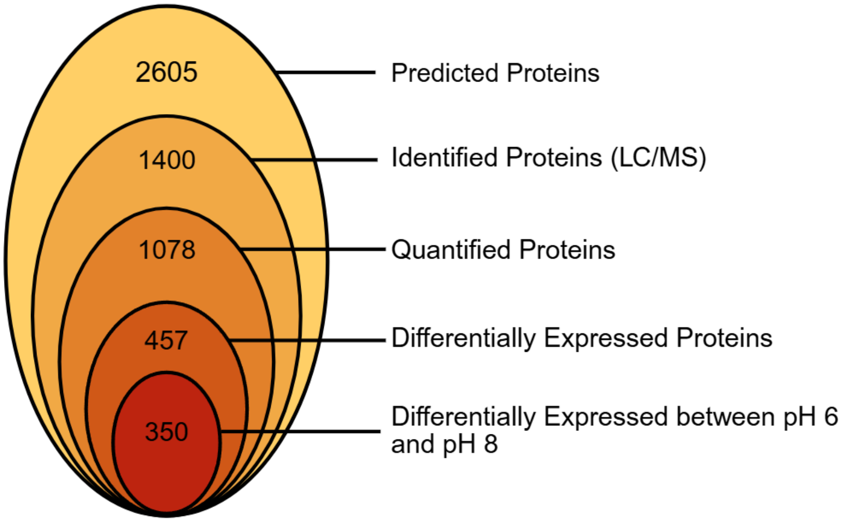

Supplement Figure S4: Proteome coverage and differential protein abundance overview for *L. lactis* FM03. Overview of the number of predicted, identified, quantified and differentially abundant proteins. In total, the chromosome and plasmids encode 2,605 predicted proteins. Of these, 1,400 were identified by LC-MS/MS, and 1,078 proteins were quantified across pH conditions. 457 proteins were differentially expressed across pH conditions (ANOVA $p \leq 0.05$), and 350 proteins were significantly different between pH 6 and pH 8 (adjusted $p \leq 0.05$).

Repair Mechanisms

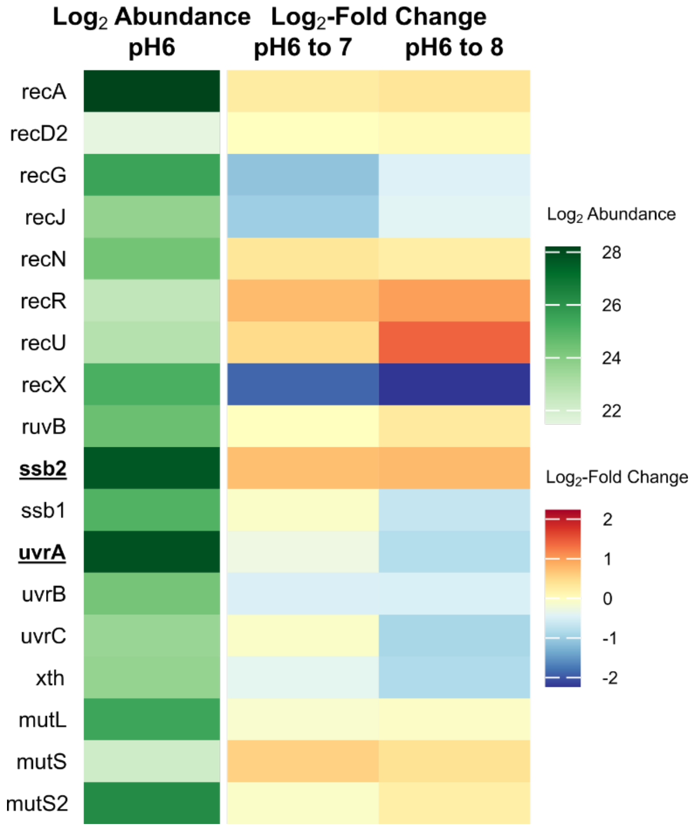

Supplement Figure S5: DNA repair mechanisms. Heatmap of proteomic changes in key proteins involved in DNA repair, showing the abundance of proteins at pH 6 (first column) and the log_2-relative fold change at pH 6 to 7 (second column) or pH 6 to 8 (third column). Red indicates higher abundance and blue lower abundance relative to pH 6. Significantly changed proteins are in bold. A complete overview of all fold-changes and *p*-values can be found in Supplement Table S2.

Ion Transporters

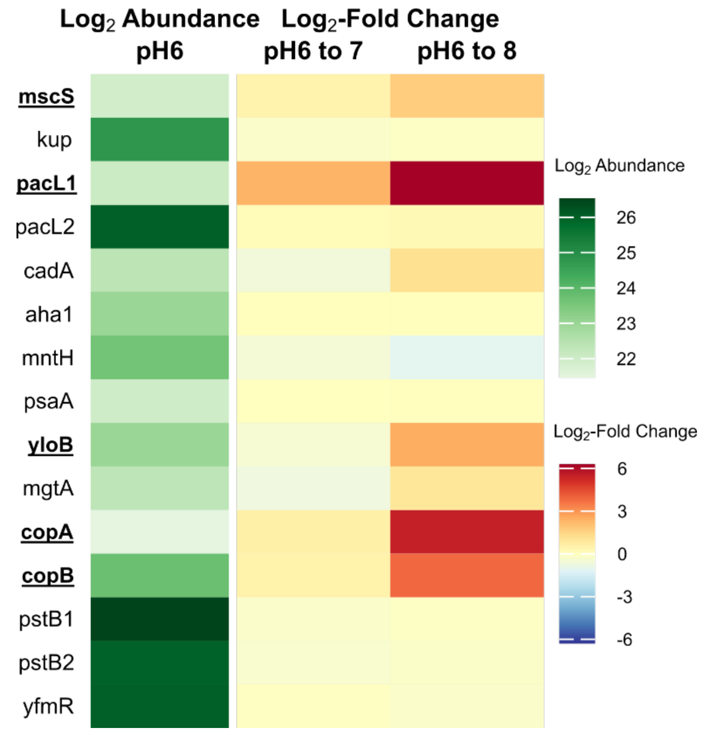

Supplement Figure S6: Cation Transporters. Heatmap of proteomic changes in key proteins involved in cation transport, showing the abundance of proteins at pH 6 (first column) and the $\log_2$-relative fold change at pH 6 to 7 (second column) or pH 6 to 8 (third column). Red indicates higher abundance and blue lower abundance relative to pH 6. Significantly changed proteins are in bold. A complete overview of all fold-changes and *p*-values can be found in Supplement Table S2.

Amino acid metabolism

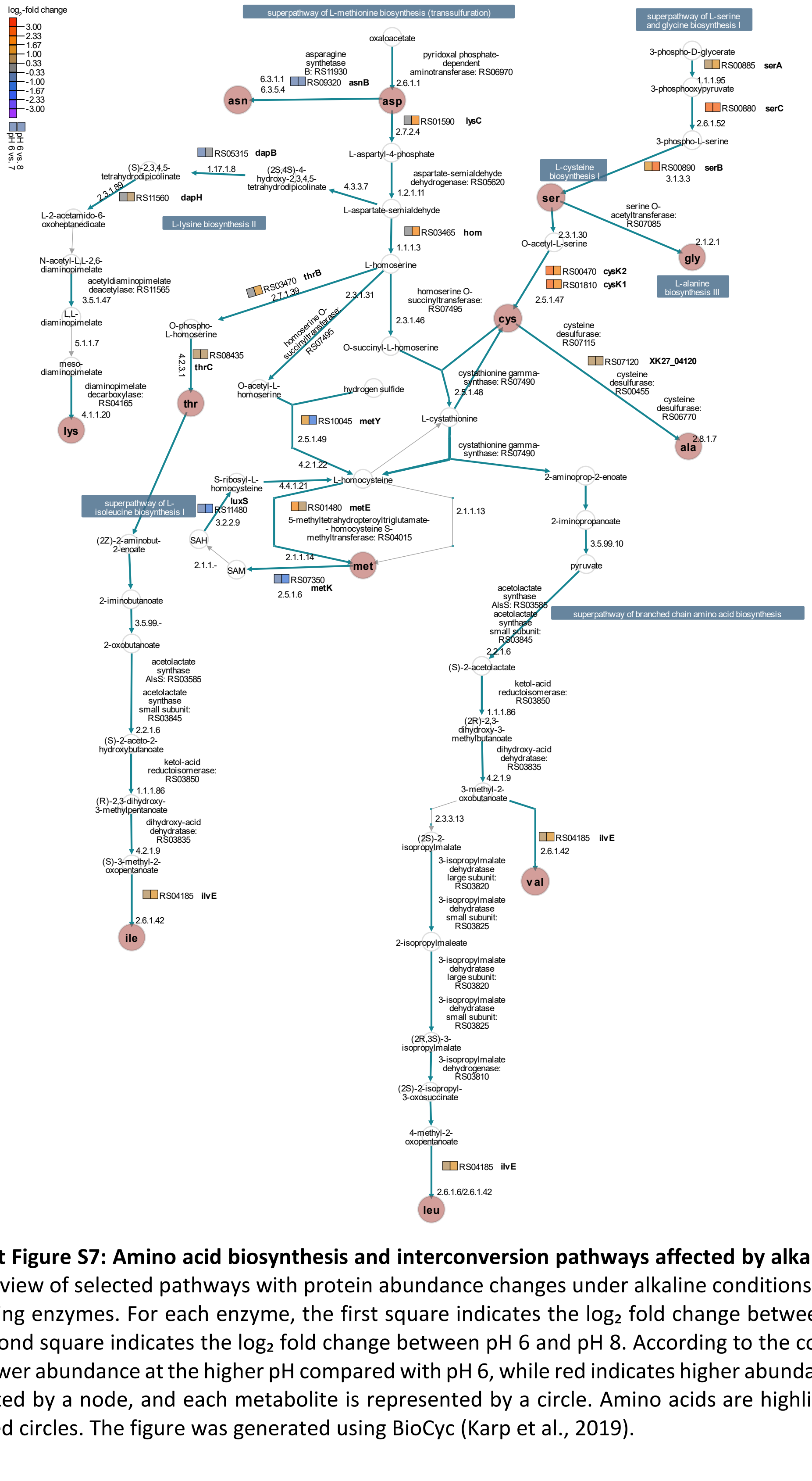

Supplement Figure S7: Amino acid biosynthesis and interconversion pathways affected by alkaline pH in *L. lactis* FM03. Overview of selected pathways with protein abundance changes under alkaline conditions mapped onto the corresponding enzymes. For each enzyme, the first square indicates the $\log_2$ fold change between pH 6 and pH 7, and the second square indicates the $\log_2$ fold change between pH 6 and pH 8. According to the colour scale, purple indicates lower abundance at the higher pH compared with pH 6, while red indicates higher abundance. Each enzyme is represented by a node, and each metabolite is represented by a circle. Amino acids are highlighted in bold and shown as red circles. The figure was generated using BioCyc (Karp et al., 2019).

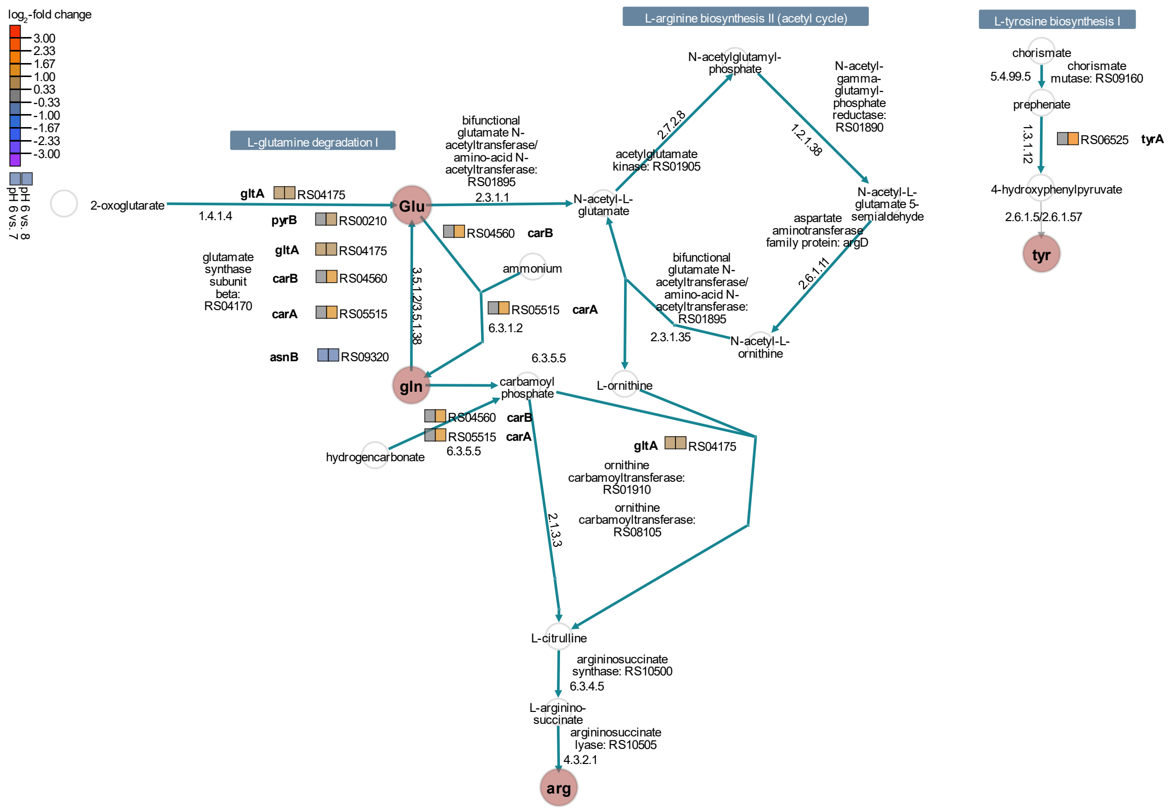

Supplement Figure S8: Amino acid biosynthesis and interconversion pathways of glutamine, arginine and tyrosine in *L. lactis* FM03 affected by alkaline pH. Overview of selected pathways involved in glutamine, arginine, and tyrosine biosynthesis and interconversion, with protein abundance changes under alkaline conditions mapped onto the corresponding enzymes. For each enzyme, the first square indicates the log_2 fold change between pH 6 and pH 7, and the second square indicates the log_2 fold change between pH 6 and pH 8. According to the colour scale, purple indicates lower abundance at the higher pH compared with pH 6, while red indicates higher abundance. Each enzyme is represented by a node, and each metabolite is represented by a circle. Amino acids are highlighted in bold and shown as red circles. The figure was generated using BioCyc (Karp et al., 2019).

References

Cantalapiedra, C.P., Hernández-Plaza, A., Letunic, I., Bork, P., and Huerta-Cepas, J. (2021) eggNOG-mapper v2: Functional Annotation, Orthology Assignments, and Domain Prediction at the Metagenomic Scale. *Molecular Biology and Evolution* **38**: 5825–5829.

de Jong, A., Kuipers, O.P., and Kok, J. (2022) FUNAGE-Pro: comprehensive web server for gene set enrichment analysis of prokaryotes. *Nucleic Acids Res* **50**: W330–w336.

Huerta-Cepas, J., Szklarczyk, D., Heller, D., Hernández-Plaza, A., Forslund, S., Cook, H. et al. (2018) eggNOG 5.0: a hierarchical, functionally and phylogenetically annotated orthology resource based on 5090 organisms and 2502 viruses. *Nucleic acids research* **47**.

Karp, P.D., Billington, R., Caspi, R., Fulcher, C.A., Latendresse, M., Kothari, A. et al. (2019) The BioCyc collection of microbial genomes and metabolic pathways. Brief Bioinform **20**: 1085–1093.
